# Scoping review of community health participatory research projects in Ghana

**DOI:** 10.1080/16549716.2022.2122304

**Published:** 2022-11-18

**Authors:** Irene A. Kretchy, Lydia O. Okoibhole, Olutobi Adekunle Sanuade, Hannah Jennings, Daniel Ll Strachan, Ann Blandford, Francis Agyei, Paapa Asante, Olamide Todowede, Mawuli Kushitor, Kafui Adjaye-Gbewonyo, Daniel Arhinful, Leonard Baatiema, Ernestina Dankyi, Carlos S. Grijalva-Eternod, Edward F. Fottrell, Ama de-Graft Aikins

**Affiliations:** a Department of Pharmacy Practice and Clinical Pharmacy, School of Pharmacy, University of Ghana, Legon, Ghana; b Institute for Global Health, University College London, London, UK; c Department of Population Health Sciences, Spencer Fox Eccles School of Medicine at the University of Utah, Salt Lake City, UT, USA; d Department of Health Sciences, University of York and Hull York Medical School, York, UK; e Nossal Institute for Global Health, University of Melbourne, Melbourne, Australia; f Department of Computer Science, UCLIC, University College London, London, UK; g Department of Family and Community Health, School of Public Health, University of Health and Allied Sciences, Ho, Ghana; h School of Health Sciences, Institute of Mental Health, University of Nottingham, Nottingham, UK; i Department of Health Policy Planning and Management, School of Public Health, University of Health and Allied Sciences, Ho, Ghana; j Faculty of Education, Health and Human Sciences, University of Greenwich, London, UK; k Department of Epidemiology, Noguchi Memorial Institute for Medical Research, University of Ghana, Legon, Ghana; l Department of Health Policy, School of Public Health, Planning and ManagementUniversity of Ghana, Legon, Ghana; m Centre for Social Policy Studies, University of Ghana, Legon, Ghana; n Department of Population Health, London School of Hygiene and Tropical Medicine, London, UK; o Institute of Advanced Studies, University College London, London, UK

**Keywords:** Developing country, diabetes, engagement, non-communicable diseases, sub-Saharan Africa

## Abstract

**Background:**

Community health participation is an essential tool in health research and management where community members, researchers and other relevant stakeholders contribute to the decision-making processes. Though community participation processes can be complex and challenging, evidence from previous studies have reported significant value of engaging with community in community health projects.

**Objective:**

To identify the nature and extent of community involvement in community health participatory research (CHPR) projects in Ghana and draw lessons for participatory design of a new project on diabetes intervention in Accra called the Contextual Awareness Response and Evaluation (CARE) diabetes project.

**Methods:**

A scoping review of relevant publications on CHPR projects in Ghana which had a participatory component was undertaken. PubMed, PsycINFO, African Journal Online, Health Source: Nursing/Academic Edition, Humanities International Complete and Google Scholar were searched for articles published between January 1950 and October 2021. Levac et al.’s (2010) methodological framework for scoping reviews was used to select, collate and characterise the data.

**Results:**

Fifteen studies were included in this review of CHPR projects from multiple disciplines. Participants included community health workers, patients, caregivers, policymakers, community groups, service users and providers. Based on Pretty’s participation typology, several themes were identified in relation to the involvement of participants in the identified studies. The highest levels of participation were found in two studies in the diagnosis, four in the development, five in the implementation and three in the evaluation phases of projects. Community participation across all studies was assessed as low overall.

**Conclusion:**

This review showed that community participation is essential in the acceptability and feasibility of research projects in Ghana and highlighted community participation’s role in the diagnosis, development, implementation and evaluation stages of projects. Lessons from this review will be considered in the development, implementation, and future evaluation of the CARE diabetes project.

## Background

Community participation has been reported as a tool for improving health through a social process where communities are empowered to identify and develop practical solutions to their health concerns [1]. We define community as a group of people with diverse characteristics who are linked by social ties, share common perspectives, and engage in joint action in geographical locations or other settings such as online [2]. According to the World Health Organization (WHO), the community participation process requires that people are enabled to actively get involved in defining their problems while taking action to achieve change [3] and the Alma Ata Declaration in 1978 identifies community participation as central to primary health care [4].

The benefits of participatory and empowering approaches for community health have been extensively reported in literature to include improved healthcare initiatives [5], improved health-related behaviours like physical activities and acquisition of new skills and greater agency over health [6–12], heightened sense of responsibility and diligence regarding health, better diffusion of health knowledge in the community and a greater use of indigenous expertise [13]. This may result in higher levels of community trust and support for locally conceived and initiated approaches [14] thereby increasing community engagement [6] and reducing morbidity and mortality over time [15].

The principle behind community participation as a tool in chronic disease research and management is that lay individuals, families, and the wider community are also producers of health, and not solely professionals in the health sector [1]. The WHO’s 1948 definition of health being ‘a state of complete physical, mental and social well-being and not merely the absence of disease or infirmity’ [3] highlights the need to approach health in a multifaceted way, understanding that improving health requires a holistic approach, beyond the exclusive insights and influence of trained health professionals. Therefore, community participation seeks to empower the community to own their challenges and develop ways to overcome them, leading to community-driven action and in concert with health professionals, policymakers, researchers, and experts in other sectors like the environment and housing[16]. Much of the work of community participation facilitation revolves around researchers building relationships with individuals who are influencers or decision-makers in the community, creating partnerships that allow for community entry, acceptance, and engagement [5]. These community-level decision makers then transfer this knowledge to others which can improve locally valued health outcomes including the sustainable management of a given disease [5].

The Community Health Participatory Research (CHPR) approach aims to equitably involve community members, researchers and other relevant stakeholders in the research process, where all partners contribute knowledge and resources and play a part in the decision-making process [6] CHPR has been applied by health researchers and practitioners to address health disparities and community empowerment for health promotion of type 2 diabetes mellitus (T2DM) [7] and other chronic disease management [14,17]. Examples of the use of CHPR include a social psychology of participation applied to ‘diagnosing’ the social reality of cardiovascular diseases and exploring the development of community-centred interventions in Accra, Ghana [8] and a participatory learning and action (PLA) intervention to address T2DM in rural Bangladesh [9]. In the Bangladesh study, there was a large reduction in the combined prevalence of T2DM and intermediate hyperglycaemia in the PLA group compared with the control group [9] and participation in the intervention and its impact were found to be equitable [10]. A participatory approach has also been successfully used in Zimbabwe, where a community-based mental health intervention, proposed by community stakeholders, resulted in an improvement in symptoms [11].

Despite these initiatives and wide acceptance of community involvement, some challenges to successful implementation have been reported. These include the complexity and meaning of the community participatory process to community members [18]. Although the community participation process can be multifaceted and challenging, drawing on lessons from previous studies can increase the likelihood of success for a community health project employing a participatory method. For this reason, research into how community participation might help in diabetes management in Ghana is relevant as it has proven to be successful in other lower middle income (LMIC) settings [9,11,12]. Ghana and other sub-Saharan African countries are facing a steady increase in the prevalence of diabetes and other NCDs, driven by the increasing incidence of NCD risk factors such as physical inactivity and unhealthy diets. This epidemiological transition is evident in Ghana, where around 43% of deaths are caused by NCDs and the health system is not currently built to tackle this rising burden [19,20]; thus the need to explore approaches that can help in the prevention and management of NCDs is crucial [19,20].

This scoping review aims to identify the nature and extent of community involvement in CHPR projects in Ghana to inform the participatory design for, the Contextual Awareness Response and Evaluation (CARE) diabetes project, with study sites in Accra. The CARE diabetes project will focus on exploring methods for examining the social context of T2DM risk, experiences and response. The CARE diabetes project will also explore how best practices in community health participation can inform our approach to data collection, analysis, dissemination, and uptake to help ensure interventions are relevant to local needs and informed by local knowledge and priorities. The CARE project focuses on T2DM, but lessons can be learned from the way other chronic NCDs are managed. Therefore, for this review, we will focus on CHPR that addresses non-communicable diseases in Ghana.

## Methods

**Figure 1.**
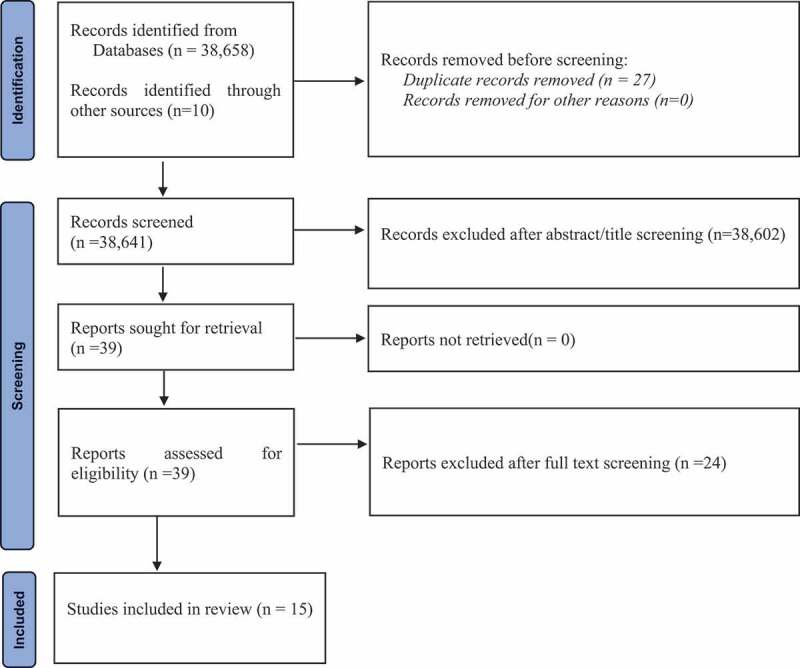
PRISMA flow diagram for screening of CHPR in Ghana [18,22].

This scoping review adopted Levac et al.’s [21] methodological framework, to map existing literature on the current state of what has been done and documented on community participatory health research projects on NCDs in Ghana [21]. This framework guided and provided clear methodological and transparent processes to our review which can be replicated. The Preferred Reporting Item for Systematic Reviews and Meta-Analyses extension for Scoping Reviews (PRISMA-ScR) [22] was used throughout the review process (screening and reporting) (see Figure 1 – Study Selection Flow Chart). The process of this review followed the first five stages of Levac et al.’s [21] methodological framework: identification of research question (stage 1); identification of relevant studies (stage 2); study selection (stage 3); data charting (stage 4); and data synthesis, collating, summarising, and reporting (stage 5).

### Search and identification of studies

Five peer-reviewed databases (PubMed, PsycINFO, African Journal Online (AJOL), Health Source: Nursing/Academic Edition, and Humanities International Complete) and one search engine (Google Scholar) were searched. In addition, three local journals (Ghana Medical Journal, Ghana Social Science Journal, and Ghana International Journal on Mental Health) were searched. The search terms used are provided in Table 1. Studies published on CHPR projects in Ghana between January 1950 and October 2021, or which had a participatory component, were included in this review and no study was excluded based on quality.

**Table 1.** Search strategy.

S1	Community health participatory research OR Community-based participatory research OR participatory research OR action research OR participatory evaluation OR action science OR collaborative inquiry OR empowerment evaluation OR community involvement
S2	NCDs OR Non-communicable Diseases OR Cancers OR Stroke OR Hypertension OR Diabetes OR Heart Attacks OR Heart Failure OR Kidney Disease OR Cardiovascular Diseases OR Chronic Lung Diseases OR Ischaemic Heart Disease OR Chronic Respiratory Diseases OR Chronic Disease OR Chronic Condition OR Myocardial Infarction OR Coronary Heart Disease OR CHD OR Ischaemic Heart Disease OR Blood Pressure OR High Blood Pressure OR Obesity OR Chronic Obstructive Pulmonary Disease OR COPD or pulmonary or bronchitis or Lung Function OR Diabetes OR Chronic Kidney Disease OR CKD OR Type 2 Diabetes OR Overweight OR Physical Activity OR Tobacco OR Tobacco smoking OR Alcohol Intake OR cholesterol OR Diabetes Mellitus OR Alcohol consumption OR Tobacco use OR Physical Inactivity OR Asthma OR Chronic Obstructive Respiratory Disease OR risk factors OR Diet OR Smoking OR mental illness
S3	Ghana OR Accra OR Greater Accra OR Kumasi OR Ashanti region OR Takoradi OR Western region, OR Cape Coast OR Central region OR Ho OR Volta region OR Koforidua OR Eastern region OR Sunyani OR Brong Ahafo region OR Tamale OR Northern region OR Bolgatanga OR Upper East region OR Wa OR Upper West region OR Bono-East region OR Techiman OR Ahafo region OR Goaso OR Savannah region OR Damongo OR North-East region OR Nalerigu OR Oti region OR Dambai
S4	S1 AND S2 AND S3
Language	English Language
Year	January 1950 – October 2021
Population	Human

### Screening and eligibility

We identified 38,658 publications and 10 additional publications from the local journals and discussion with an expert in CHPR (AdGA). A total of 27 duplicates were removed and the remaining 38,641 publications were retained for subsequent screening for eligibility. After title and abstract screening, a total of 38,602 were excluded because they did not focus on NCDs and did not use a CHPR approach. Studies that were published in the English language, focused on NCDs, were conducted in Ghana and used a CHPR approach were included in the review. A total of 39 publications met the inclusion criteria. The full text of the 39 publications were then retrieved for detailed review. After the full text review, only 15 studies were found to have used the CHPR approach, and are therefore included in the synthesis.

### Extent of community participation

The extent of community participation was assessed using Pretty’s [23] participation typology as adapted by Snijder et al. [24] and Wagemakers et al. [25]. This typology describes seven levels of community participation which range from no participation (i.e. completely top-down approach from outside actors) to self-mobilisation (i.e. completely bottom-up approach from the community where the project is situated). As community engagement can vary during the lifetime of a project, we assessed the level of community engagement separately for four phases of the project: diagnosis (identifying a community’s priorities); development (of appropriate strategies to address the priorities); implementation (of the strategies); and evaluation (of the effectiveness of the project) [24]. Definitions of the seven levels of community participation in the four phases of project development are provided in Appendix 1.

For the 15 eligible studies, the level of community participation was assigned a score between 1 and 7 for each of the phases of project development. These are summarised as follows: no participation (score 1); passive participation (score 2 – meaning the community was only informed about the project); participation by information (score 3 – meaning information was collected from the community without their participation and without providing feedback); participation by consultation (score 4 – meaning information was collected from the community, feedback was given and further inclusion of community was sought); functional participation (score 5 – meaning community collaboration, but on outsiders’ terms); interactive participation (score 6 – meaning collaboration on mutually defined terms); and self-mobilisation (score 7 – meaning outsider’s work in community was based on community’s terms) [24]. The scoring was carried out independently by two research team members (PA and FA). Scores were given based on both reviewers reaching a mutual understanding of the typology. Results were discussed for inter-rater reliability and disagreements were resolved during a team meeting. The overall scoring was also reviewed by a third team member (LO) for triangulation. Where studies did not have enough information to assess all phases of project development, they were marked as ‘unknown’.

## Results

### Study characteristics

Fifteen studies were included in this review; all the included studies were published between 2006 and 2021. Community Health Participatory Research (CHPR) in Ghana has been conducted by researchers from multiple disciplines including public health, global health, epidemiology, population science, economics, psychology, and anthropology, based on the affiliations of the authors. Study sample sizes ranged from twenty-seven (27) [26] to two thousand four hundred (2400) participants [27]. Three studies did not provide the sample size (Appendix 3) [8,28,29]. Participants included a range of stakeholders in their studies – community health workers, patients, caregivers, policy makers, community groups, service users and providers.

Table 2 provides a summary of study characteristics. Studies were concentrated in nine regions, the majority being in the Greater Accra region (5).^1^^1^As of 2020, there are 16 regions in Ghana. Prior to this and at the time the majority of the studies were conducted, there were 10 regions in Ghana. This may explain why studies were not retrieved for some of the new regions. Three studies were conducted in rural areas, six in urban areas, four in both urban and rural, and two in peri-urban sites. A map highlighting the regions can be found in Appendix 1. Twelve studies focused on NCDs, and three on general healthcare/quality of care. Studies adopted several methods, including mixed methods. Ten studies used quantitative methods within the broader project while ten adopted qualitative approaches.

**Table 2.** Summary of study characteristics.

Summary of study characteristics		Frequency
Region	Greater Accra	5
	Eastern	2
	Upper East	2
	Upper West	1
	Ashanti	1
	Brong Ahafo	1
	Western	1
	Greater Accra and Bono East	1
	Northern, Upper East and Upper West	1
Focus of study	Hypertension	4
	Cardiovascular disease	3
	Mental health/depression	2
	General primary healthcare	2
	Stroke	2
	Quality of healthcare/healthcare delivery	2
	Obesity and cardiometabolic disease	1
Study site	Urban	6
	Urban and rural	4
	Rural	3
	Peri-urban	2
Study designs	RCTs	3
	Cross-sectional	3
	Mixed methods	3
	Prospective cohort studies/longitudinal	3
	Case study	2
	Quasi-experimental	2
	Implementation science study	1
	Ethnography/participatory action	1
Research themes	Disease management and control	12
	Health promotion and prevention	3

Studies have been divided into two key themes – ‘Health promotion and prevention’ and ‘disease management and control’. ‘Disease management and control’ includes studies that focus on primary healthcare, treatment and interventions that are primarily focused on managing existing conditions. ‘Health promotion and prevention’ focuses on health promotion activity including education, screening, and interventions to prevent disease or promote healthy behaviours.

### Extent of community participation

Table 3 summarises the level of community participation across the four phases of project development. Table 4 summarises the number of studies relevant to each of the seven levels of community participation across the four phases of project development. The highest levels of participation (level 5 to level 7) were found in the diagnosis phase for two studies (13.3%) [30,31], in the development phase for four studies (26.7%) [8,29,30,32] in the implementation phase for five studies (33.3%) [8,29,30,32,33] and in the evaluation phase for three studies (17.6%) [31–33]. Overall, levels of participation were highest in one of the studies on healthcare quality, although evaluation was scored as ‘unknown’ [30].

**Table 3.** Level of community participation in each phase of project development for each study.

First author (Year)	Focus of study	Four phases of project development			
		Diagnosis	Development	Implementation	Evaluation
Adler et al. (2019) [32]	Hypertension	3	Unknown	5	5
Adler et al. (2020) [33]	Hypertension	2	5	6	5
Adongo et al. (2014) [29]	Community-based Health Planning and Services (CHPS)	4	5	6	1
Agongo et al. (2021) [31]	Obesity and cardiometabolic disease	5	3	3	4
Alhasan, et al. (2016) [30]	Quality healthcare	6	5	5	Unknown
Appiah et al. (2020) [43]	Depression	2	2	2	1
Baatiema et al. (2013) [26]	Community participation^2^	2	2	2	5
(Cappuccio et al. 2006) [34]	Hypertension	2	1	2	3
de-Graft Aikins (2014) [39]	CVD	4	4	4	4
de-Graft Aikins (2020) [8]	CVD	4	6	6	3
Gaala (2008) [28]	Health delivery and management	2	2	3	4
Haykin et al (2020) [40]	CVD	2	1	1	2
Lamptey (2017) [27]	Hypertension	2	1	2	2
Ojo et al. (2020) [42]	Stroke	2	2	3	3
Read et al. (2020) [41]	Mental health	4	4	4	4

Adapted from Snijder et al.,[24]. Possible scores range from 1 to 7: 1 = no participation; 2 = passive participation; 3 = participation by information; 4 = participation by consultation; 5 = functional participation; 6 = interactive participation; 7 = self-mobilisation, UNK = unknown.

**Table 4.** Number of studies across the levels of community participation and phases of project development.

Seven levels of community participation	Four phases of project development			
	Diagnosis	Development	Implementation	Evaluation
1. No participation	-	3	1	2
2.Passive participation	8	4	4	1
3.Participation by information	1	1	3	4
4.Participation by consultation	4	2	2	4
***Least active involvement sub-total (levels 1–4)***	**13**	**10**	**10**	**11**
5. Functional participation	1	3	2	3
6. Interactive participation	1	1	3	-
7. Self-mobilisation	-	-	-	-
***Most active involvement sub-total (levels 5–7)***	**2**	**4**	**5**	**3**
Unknown	-	1	-	1
Total	15	15	15	15

The participation of the community was described with insufficient detail to be assessed (unknown category) for one study in the Development phase (5.8%) [33] and one (5.8%) in the Evaluation phase [30]. Overall, levels of community participation across all studies were assessed as low. Most studies scored between 1 and 4, the least active levels of involvement, and no studies were identified at level 7 (self-mobilisation) (Table 4).

### Aims and outcomes of the studies

A summary of the studies’ aims, and outcomes is provided in Table 5. Eleven studies reported a positive impact of community development projects on health and wellbeing of the studied participants, particularly those where community participation was high. A detailed description of all papers, including study design, can be found in the supplementary material (Appendix 3).

**Table 5.** Aims and outcomes of studies.

First author (year)	Study design	Project aim	Outcomes of study
Adler et al. (2019) [32]	Prospective cohort study; Quantitative	Evaluated the effectiveness of ComHIP for controlling hypertension in patients who were enrolled in the programme.	Enrolled participants were screened and those with hypertension were followed for at least a year. Of those screened, 72% of participants had their hypertension under control and there was also a reduction in blood pressure. Overall, it found that those in ComHIP had improved blood pressure control. Change in knowledge of risk factors was not measured due to low retention. Participants who enrolled earlier were much more likely to stay in the programme than those who enrolled later.
Adler et al. (2020) [33]	Qualitative; individual interviews and focus group discussions	Analysed the barriers and facilitators of the main components of ComHIP	The effectiveness of the intervention was yet to be determined but overall, patients and nurses reported positive experiences within ComHIP and that it helped them manage their hypertension.
Adongo et al. (2014) [29]	Experimental (quasi-experiment design)	Evaluated whether the design and implementation of CHPS are transferable to an urban setting.	Implementation activities that worked in a rural setting were not completely transferable to an urban poor settlement setting.
Agongo et al. (2021) [31]	Qualitative study: interviews, focus groups and durbars	Described the processes of community engagement, challenges encountered, and major lessons learned during the AWI-Gen study	Participants appreciated the feedback and information they received because of the study. However, challenges included the participants frustration at the time it took to receive feedback. There were also issues with the location and time of year the study was conducted. Key recommendations were provided.
Alhassan, et al. (2016) [30]	Randomised Control Trial	Designed and implemented systematic community engagement interventions using existing community groups already engaged in healthcare quality assessment.	The systematic community engagement concept is a complementary quality improvement tool to improve the experiences of clients and the client-healthcare provider relationship. It was also deemed to be cost-effective, community-focused, and sustainable
Appiah et al. (2020) [43]	Quasi-randomised controlled trial	Examined the effectiveness of the Inspired Life programme (a positive psychology intervention) in promoting positive mental health and reducing the symptoms of depression in a sample of rural poor adults in Ghana.	Participants were assigned to an intervention or control group as part of the inspired life programme, and it found that there was a greater improvement in positive mental health with a reduction in symptoms of depression in the intervention group immediately after the intervention and 3 months after.
Baatiema et al. (2013) [26]	Qualitative – IDIs, FGDs, community conversations	Assessed participation in CHPS programme in Upper West region of Ghana	Participation was sustained by using community resources, making use of existing CHPS integration with community structures and by aligning CHPS services with the community’s interests.
Cappuccio et al. (2006) [34]	Cluster randomised trial	Aimed to reduce salt intake and blood pressure in rural Ghana through health promotion and education.	A health education programme for all villagers (with an intervention and control group) found that there was a significant positive relationship between salt intake and systolic and diastolic blood pressure. There was a reduction in blood pressure compared to the control group.
de-Graft Aikins et al. (2014) [39]	Longitudinal design; Mixed-method – survey, interviews, focus group discussion	Discussed the impact of CVDs on primary healthcare services in urban poor communities in Accra and reviewed changes in universal health coverage in Ghana and the role of CHPS and the National Health insurance scheme in Primary Healthcare.	Participant’s health measurements were taken, and a qualitative survey conducted to gather baseline and long-term data on community knowledge, experiences and responses to CVD. There were also focus group discussions and interviews with stakeholders. The authors concluded that the rising burden of NCDs in Ghana will be a challenge in relation to achieving universal health coverage stating that CVD is not ‘accessible, equitable or responsive’ to the needs of communities.
de-Graft Aikins et al. (2020) [8]	Participation approach, mixed method data – qualitative interviews, household surveys, GIS mapping	Describes the conceptual, methodological, and practical insights from a longitudinal social psychological project that aims to build cardiovascular disease (CVD) competence in a poor community in Accra.	Medical and self-care practices were monitored throughout the study and preliminary data found that the intervention had ‘moderate’ impact on blood pressure and blood glucose and on positive lifestyle changes such as healthy eating. The intervention also identified poverty, lack of men’s participation, the political economy, and concerns around the sustainability of the project as challenges to successful implementation of the intervention.
Gaala (2008) [28]	Case study; Qualitative – focus group, interviews	Explained the different types of community participation in the public health sector to identify the ways communities take part in healthcare delivery and management.	Community participation is used for improving the health status of communities as opposed to building confidence or empowering communities.
Haykin et al. (2020) [40]	Implementation science study; Qualitative – in-depth individual interviews	Aimed to develop a protocol for CHPS nurses to provide CVD care using the WHO-PEN protocol by allowing nurses and nurse supervisors to identify factors that constrain or facilitate CVD screening and treatment.	Three key themes were identified: ‘community demand for CVD care; community access to CVD care; and provider capacity to render CVD care’. The authors concluded that a trial of this intervention is needed to assess the impact on CVD risk factors.
Lamptey et al. (2017) [27]	Quasi-experimental design	This study was an evaluation of a ComHIP. It presents the findings of a base-line cross sectional survey which focused on hypertension prevalence, awareness, treatment, and control.	After evaluating the ComHIP programme, through surveys and analysing anthropometric and clinical data from participants, researchers found that hypertension prevalence was high in the study population, as was awareness, but there were very low levels of hypertension treatment and control.
Ojo et al. (2020) [42]	Multi-method qualitative study – focus group discussion and key informant interviews	This study aimed to adapt a skills-based stroke prevention intervention for communities by capturing the perceptions of various stakeholders in Urban Ghana using the Discharge Education Strategies for Reduction of Vascular Events (DESERVE) intervention as a framework.	The DESERVE intervention was found to be a good fit across all the stakeholders and certain elements of the intervention were deemed transferable in urban settings.
Read et al. (2020) [41]	Qualitative study; ethnographic research; participatory research, naturalistic observations, and in-depth interviews	Evaluated the current policy context around mental health and human rights in Ghana and evaluates the challenges around implementing legislation to protect the right of those with mental illness.	Community participation, through focus groups, interviews and observation was used to identify the experiences of those with lived experiences of mental illness and other relevant stakeholders. Participants encountered significant social challenges due to their mental health. The existence of potential human rights legislations such as the Disability Rights Act, Labour Law and Mental Health act are important for the active recovery and social inclusion of individuals living with mental illness.

## Discussion

The purpose of this scoping review was to identify existing knowledge on and the extent of community involvement in participatory health research projects in Ghana. This was done to understand how best practices in community health participation can inform approaches to project design and implementation. A total of 15 studies were identified and reviewed. Most of the included studies had some level of community participation, although the extent of this participation varied. The review also showed that levels of community participation were dependent on the type of project, the health condition being explored, and the study design. Regardless, the findings of this review suggest that CHPR projects in Ghana were largely successful, whereby studies reported that community participation is a promising approach to improve the well-being and health of a community.

Community participation has been identified as a key element of building relationships and strengthening people centred primary healthcare [4]. Community participation in health offers many advantages such as empowering the community, ensuring their needs are met, and ensuring that strategies and methods are culturally and socially acceptable. In addition, this community participation approach can give the community a sense of responsibility for their health and well-being [35,36]. Research shows that early involvement of participants in the development of a project leads to better design, targeted benefits, more equitable distribution, and greater emphasis on the community itself [37].

### Levels of community participation in the phases of community development

Several trends were identified in relation to the involvement of participants in the identified studies. As mentioned, most of the identified studies involved low levels of participant engagement. Overall, the greatest level of involvement in the phases of project development, as determined by our scoring [24], was the implementation stage of the study where four studies [29,30,32,33] scored between 5–7. The stage where there was the least active participation was the diagnosis stage. This may be because many of the studies were developed in response to funding calls suggesting that the aims/objectives of the study would have already been defined prior to community engagement.^2^^2^Although not a traditional health determinant, this study explores the community response to local health services which can have an impact on health.

The two studies [29,32] that scored highest in the implementation stage showed a significant change or improvement in the programmes they implemented or identified areas for improvement. For example, Adongo et al.[29] found that the CHPS model was not transferable from a rural to urban setting. This learning is incredibly important, particularly in relation to implementing an intervention in a community setting. Recognising that local cultural and community context matter during intervention implementation helps researchers to prioritise this during the design of an intervention. Engaging with the community, involving them in the research process and having their support can determine whether a project is successful or not, as has been demonstrated in existing reviews [24,35–37].

The lack of studies with the highest level of participation (level 7) may be a result of existing structures around research formulation and development. Many research projects are developed in response to funding opportunities, and objectives are decided by researchers and relevant stakeholders, such as funders, ahead of time, therefore making it less feasible for the community to be involved in the ‘diagnosis stage’ or for studies to reach the most active levels of participation. It could also be said that many projects would not occur without the input of those with the time, skill, and commitment to the research, who are likely to be outsiders to the community [38]. Although it is important to involve the community as much as possible in CHPR, prioritising more active research, from functional participation to self-mobilisation, may be a more feasible way of balancing ‘outsider’ interests, with participation from the community.

#### How participation was achieved

As indicated earlier, the level and type of participation varied between studies. In several studies, community participation was achieved through individual interviews (semi-structured or in-depth) and focus discussion groups with the community, often before the intervention was developed or implemented [39–42]. Also, key informant interviews, pilot studies, qualitative appraisal consultations and community meetings were held to engage with the community. Reasons for engaging with the community, as stated in some of these studies, include: 1) to understand cultural acceptability 29; 2) to understand geographical and cultural boundaries [26] 3) to test and explore key concepts [8,43]; 4) to test the feasibility of a particular approach [8,29]; and 5) to involve the community in the development of an intervention or test the acceptability of the proposed study [8,26].

An important finding was that in some of these studies, particularly the studies conducted in rural areas, community leaders such as chiefs and elders and lay community members were approached prior to the commencement of the studies [29–32,42]. This is known as community entry, the process of initiating, nurturing, and sustaining a relationship with the community, particularly its leaders, to secure and sustain the community’s interest [44]. In several studies, community durbars were also mentioned. Durbars are ‘formal community-wide gatherings that include cultural activities such as drumming and dancing and provide an opportunity for information to be shared with a large number of people simultaneously’ [45]. Durbars are particularly relevant in rural settings. In one study, researchers used these gatherings to give community members the opportunity to express their concerns and views on the study prior to research being carried out as well as after research had been conducted [31]. It allowed researchers to mobilise support but also shape their study according to the opinions of the community in the study area. This finding is important to note and suggests that involving a range of community members and key stakeholders in the early stages of a project is beneficial, shows respect for traditions and customs and demonstrates a willingness to work with the community, encouraging support, which in turn may promote participation.

In the same study [31], where activities were conducted at a chief’s palace, a key finding was that not all community members felt comfortable expressing their opinions and this was mentioned in other papers [8]. This is also important as it demonstrates the need to consider power dynamics and incorporate the involvement of marginalised groups when conducting research in a community context. Regardless, community leaders are important as research partners as they ensure broader community acceptability, build trust, and establish researchers’ credibility. Community leaders such as chiefs are often viewed as ‘important representatives of community interests and key gatekeepers’, particularly in the African context [31,35]. This approach has even been incorporated into Ghana Community-Based Health Planning and Services Initiative policy as the standard for community engagement in Ghana [46]. This being adopted by several authors of papers included in this review and in Ghanaian health practice, makes it clear that it is a key element of successful community participation in Ghana.

## Limitations

Several limitations have been identified. This review aimed to identify the breadth of information available on CHPR in Ghana as opposed to the depth so further analysis was not conducted. It was also a rapid review so relevant studies may not have been included. Studies were mainly identified through database searching so studies not published or available online would not have been included and paper did not undergo critical appraisal as this was beyond the scope of this study. It is also important to note that although no studies were identified as having the most active participation (level 7), research that is entirely community led may not be feasible in the context of these projects and scoring for this review, although standardised, was subjective to the reviewers. Regardless, the lack of studies with active participation (levels 5–7) makes it clear that research encouraging more active participation (levels 5–7) from the community is necessary. There is also opportunity to engage with donors regarding the value of early and sustained community participation to project effectiveness and sustainability given that funders’ requirements can act to prevent such participation.

## Conclusion

Despite the variability in the studies, there are many positive examples of community participation in Ghana that highlight the benefits of involving the community in the various stages of project development. All studies largely demonstrated that community participation plays an important role in the acceptability and feasibility of a study. However, it is important to note that studies are specific to their context, whether that be rural, urban, or targeting a specific health condition or behaviour. Therefore, applying these findings in an external context may be limited, yet important lessons can be learned from this approach.

Following on from this review, we believe there is a greater need for research focusing on more active participation from communities in Ghana including those affected by NCDs. This research should be largely led and determined by the community. This should be at all stages, from diagnosing the issue to designing, implementing and evaluating the study. There is also a need for more early engagement in projects as most participation was found to be in the later phases of projects, and it is clear researchers would benefit from working with community leaders and elders as it has proven to be effective. For this to be done, more funder commitment to implement research that prioritises community participation, particularly in the early stages of research, is necessary. In thinking through and planning for the CARE project, the team has actively incorporated the above lessons in the development, implementation, and future evaluation of the project.

## Supplementary Material

Supplemental Material

## Paper context

Community participation in health research where communities are empowered to identify their problems and develop practical solutions is reported as an essential tool for improving health care. This review aimed at synthesising evidence on community health participatory research projects in Ghana, to guide the design and implementation of a participatory design of a new project on understanding and responding to the burden of diabetes in Accra. Early engagement from project diagnosis to evaluation is recommended.
