# Supplementary material for: Scoping review of community health participatory research projects in Ghana
Source: Glob Health Action. 2022 Nov 18;15(1):2122304. doi: 10.1080/16549716.2022.2122304 (PMC9677985; doi:10.1080/16549716.2022.2122304)
Supplement: Supplemental Material [file ZGHA_A_2122304_SM2694.docx]

**SUPPLEMENTARY MATERIAL**

Appendix 1


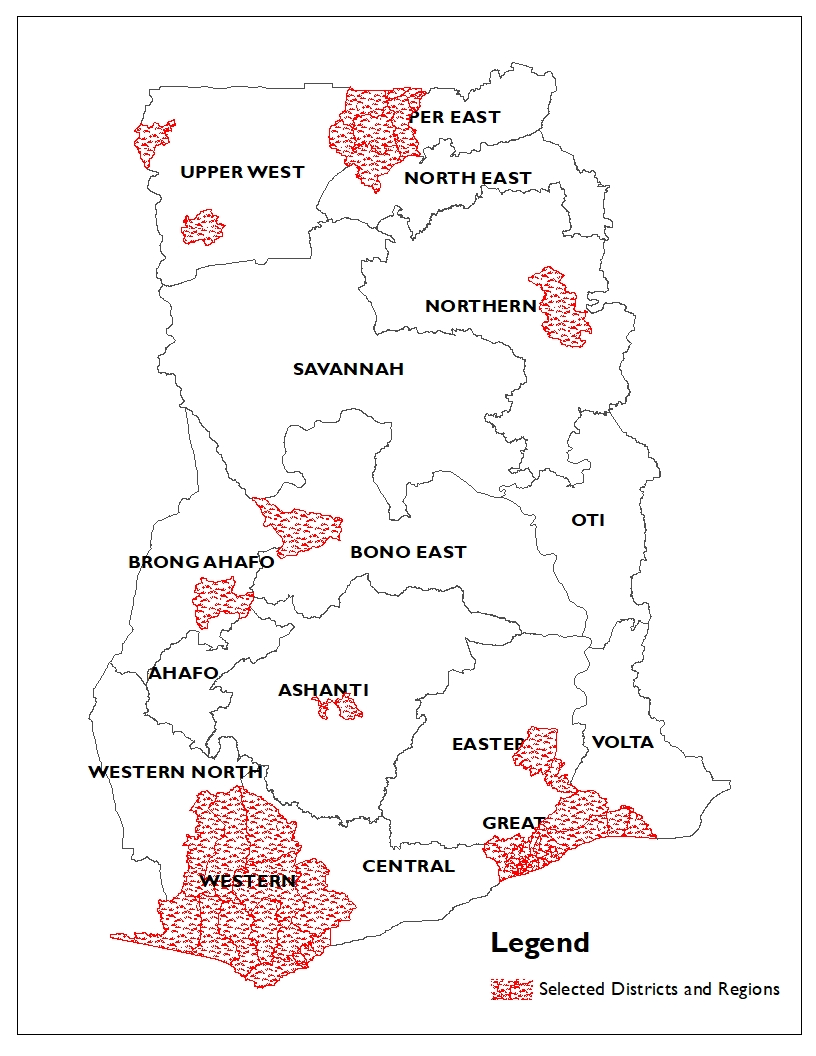


***Figure 1 CHPR locations in Ghana***

Appendix 2

**Table 1 Definitions of the seven levels of community participation in the four phases of project development**

| **Seven levels of community participation** | **Four phases of project development** | | | |
| --- | --- | --- | --- | --- |
|  | **Diagnosis** | **Development** | **Implementation** | **Evaluation** |
| ***1. No participation*** | Completely top-down, community is not informed about or asked about issues in their community. | Top-down, community is not informed about the development of the project. | Top-down, community is not informed about the implementation of the project, only about activities they’re involved in | Top-down, community receives no information about evaluation. |
| ***2. Passive participation*** | Outsiders decide on the issues that need to be addressed, community is informed. | Outsiders control development, community is informed, but has no input. | Outsiders control the implementation, community is informed, but has no input. | Outsiders control the evaluation, community is informed, but has no input. |
| ***3. Participation by information*** | Outsiders have control, community participates by providing information about their community.  No feedback to the community and no checking for agreements | Outsiders have control over development, community potentially provides information about what they want, but outsiders don’t necessarily respond to this. | Outsiders control implementation, community might provide information useful for implementation, but outsiders don’t necessarily listened to this. | Outsiders control evaluation, community provides information through surveys and/or interviews, focus groups.  Findings are not shared or checked for accuracy. |
| ***4. Participation by consultation*** | Outsiders define problems and consult with community about their agreement, using outsider defined processes. | Outsiders consult with community about potential projects to develop, but outsiders make final decision. | Community participates in activities decided upon by the outsiders | Outsiders define evaluation process, community provides information and might make suggestions for improvement and feedback provided |
| ***5. Functional participation*** | Outsiders have predetermined goals and community assists in defining issues within those goals, outsiders make final decisions. | Community works together with outsiders to develop projects decided upon by the outsiders. | Community and outsiders work towards implementation of projects, based on outsiders’ goals and processes. | Community and outsiders work together in evaluation, based on goals as set by the outsiders. |
| ***6. Interactive participation*** | Outsiders and community work together to identify the issues in the community and set goals for the project. | Outsiders and community work together to develop suitable projects to address the agreed upon goals. | Community and outsiders implement the developed projects together, community has control and uses local resources. | Evaluation methods are decided upon together and conducted in partnership. |
| ***7. Self-mobilisation*** | Completely bottom-up, community identifies their own issues and sets their own goals, might contact outsiders to assist them where needed. | Bottom-up, community makes decisions about project development, apply for funding and potentially contact outsiders where needed. | Community implements projects, contacts outsiders for resources where needed, but remains in control over resources. | Community conducts evaluations potentially contacts outsiders for assistance but stays in control over evaluation. |

Source: adapted from Pretty (1995) [23] Wagemakers et al. (2008) [25] and Snijder et al. (2015) [24].

| **Appendix 3** | | | | | | | |
| --- | --- | --- | --- | --- | --- | --- | --- |
| **Authors, pub year** | **Community/hospital, region** | **Study locations (rural, urban)** | **Focus (**e.g. NCDs, infectious diseases, etc) | **Study designs** | **Recruitment strategy**  **(e.g** community, churches, clinics**)** | **Sample size**  **&**  **age range** | **Key findings** |
| **Adler et al. (2020)** | Manya Krobo, Eastern Region | Peri-urban | NCDs: hypertension | Cross sectional, Qualitative [FGDs] | Clinic | n=55  Patients [n=31]  Healthcare providers[n=10]  Licenced chemical sellers[n=7]  Policy makers[n=7]  Age range [not provided] | - The Community Health Improvement Project [ComHIP] increased hypertensive patients’ awareness which helped them to monitor their blood pressure. - Healthcare workers including nurses and chemical sellers indicated an increase in the knowledge of risk factors, prevention and treatment of hypertension after training. - The program improved interaction between patients and their health workers. - All nurses expressed their frustration for their inability to dispense antihypertensive medications to patients. - ICT enhance management of hypertension through SMS and CommCare for keeping patients’ records. - Healthcare professionals expressed doubt about the sustainability of the program. |
| **Adler et al. (2019)** | Manya Krobo, Eastern Region | Peri-urban | NCDs: hypertension | Prospective Cohort study,  Quantitative [cross sectional] | Community | n=1339  Age range  [30-44] n=240  [45-54] n=314  [55-64] n=365  [65+] n=420 | - Participants who remained in the program for 6 or 12 months shoed a reduction in their Diastolic and Systolic Blood Pressure]. - The program also led to hypertension control from under half to more than two-thirds among participants. - Factors including age, education, hypertension under control at enrolment and enrolment date were associated with retention in the program for 12 months. |
| **Adongo et al. (2014)** | Ga East Municipality, Greater Accra Region | Urban | Primary healthcare services | Experimental [quasi-experiment design] | Community | No sample size and range provided | - Findings indicate that there are distinct contextual differences that exist in urban and rural communities which make it difficult in transferring CHPS model directly to urban settings. This includes a lack of organisational structure, centralised traditional leadership, non-availability of land and limited volunteering  in urban settings,  the need to focus more on adolescent and health needs in addition to chronic diseases and sanitation in urban settings. |
| **Alhasan, et al (2016)** | Greater Accra and Western Regions | Urban and Rural | Quality of healthcare | Randomised Control Trial | Clinics | Health facilities [n=64]  Facilitators [n=52]  Community groups [n=52]    Age range  [18-30] =35%  [31+] =65%    **N= 168** | - Non-technical areas that were rated lowest among community groups include provision of information to clients, drug availability, directional signs, fairness in queueing, waiting times and use of suggestion boxes. - Community groups perceived services provided in private health facilities to be better than public health facilities. - Community groups dominated by artisans and elderly members (60+ years) had better perspectives on healthcare quality than youthful groups. - The second round of assessment showed improvement in the perceptions of healthcare quality across all indicators. |
| **Baatiema et al (2013)** | Wa Municipal, Upper West Region | Urban | Community participation, Community-based health planning and services [CHPS] | Cross sectional  Qualitative [IDIs and FGDs] | Community | Total sample [n=17]  Service users [n=12]; Mean age =42    Service providers [n=3]; Mean age =52    Community health committee members[n=2]; Mean age =50    N= 34 | - Factors which facilitated community participation included community mobilisation of local resources to support  CHPS through cash and kind contributions; integration of CHPs with pre-existing community structures such as unit committees, health volunteers and traditional birth attendants; lack of external interference from health professionals. - Factors which undermined community participation in the CHPS program include the dominance of males and undemocratic community leadership and management style. |
| **Cappuccio et al (2006)** | 12 communities in Ejisu-Juabeng and Kumasi districts, Ashanti region | Rural and semi-urban | NCDs, Hypertension / health promotion | Study design: community-based cluster randomized trial  Methods: Quantitative – survey, measured height, weight and BP | Community | Sample size = 1,013 participants  Mean Age = 55 years | - Intervention communities received health promotion on reduction of salt intake while control group received nothing. Dependent variables measured were urinary sodium excretion (UNa) and BP levels - At baseline, there was positive relationship between salt intake and both systolic and diastolic BP - After six months, the intervention group had significant reduction in systolic and diastolic BP compared to the control communities - There were no significant differences in urinary sodium between intervention and control communities - When time of day was controlled for, there was significant positive relationship between change in UNa and systolic BP, regardless of randomization, suggesting that lower UNa, the lower the BP |
| **de-Graft Aikins et al (2014)** | Ga-Mashie, Greater Accra region | Urban | Focus: hypertension and diabetes, Management and control / task shifting | Study design: Longitudinal design  Methods: Mixed-method – survey, interviews, focus group discussion | Community | 32 health workers  Age range = 20 – 60 years | - Primary healthcare delivery to the communities is characterized by pluralistic medical system; government polyclinic, private health centres, pharmacies and chemical shops, traditional herbal shops and religious spaces (churches, mosques) that offer faith-based healing. - Individuals living with hypertension and diabetes perceived all the services as legitimate providers of care and therefore regularly used, except traditional shrines - Feasibility assessment of task-shifting for CVD care among community health workers (CHW) showed that the CHWs lacked basic knowledge of CVD risks, prevention, control and treatment - Huge gaps in knowledge of CHWs were identified in areas of association between  read meat consumption and poor health, diabetes as risk factor for heart diseases and association between family history and risk of heart disease, and the lifelong use of BP drugs for individuals living with hypertension |
| **De-graft Aikins (2019)** | Gamashie, Greater Accra region | Urban | NCDs, Cardiovascular disease | Participation approach, mixed method data - qualitative interviews, household surveys, GIS mapping | Community | Not stated | - There is a strong tendency to focus solely on the disabling effects of poverty, social exclusion, and marginalisation when conducting research in poor communities. - Many poor communities, like Ga Mashie, have complex and dynamic histories and forms of capital and agency that structure and guide their internal and external relations. - Medical and self-care practices were monitored throughout the study and preliminary data found that the intervention had ‘moderate’ impact on blood pressure and blood glucose and a had impact on positive lifestyle changes such as healthy eating. The intervention also identified several challenges to successful implementation of the intervention. |
| **Gaala (2008)** | Yendi, Builsa and Lawra districts, Northern, Upper East and Upper West regions respectively | Rural and urban | Surveillance, control/prevention and management | Study design: Case study, qualitative – focus group, interviews | Community | Not stated | - Community participation was found to be limited in terms of needs assessment and program planning - There was no consensus regarding the scope of curative health service delivery within which the communities could participate. - Participation in curative health services by auxiliary staff of the communities was the most contentious. There was divergent opinions and division of public health professionals concerning the aspect of participation due to the fact that the professionals are unable to monitor the community auxiliary staff. - The nature of participation of the communities was determined by the needs and interests of the community members - Community participation in health is more as means rather than end process towards improvement of health of the communities rather than just building the confidence and empowering of the communities. - Partnership and collaborative arrangements are fundamental to the communities’ participation |
| **Haykin et al (2020)** | Kasena-Nankana East and West districts, Upper East Region | Rural | CVD/NCDs, screening, care and treatment | Study design: Implementation science study  Method: Qualitative - interviews | Community | Sample = 31  Age = not stated | - Three thematic factors were found to constrain or facilitate CVD screening and treatment; (i) community demand for CVD care, (ii) community access to CVD care, and (iii) provider capacity in delivering CVD care - The nurses and the supervisors indicated that prevalence of risk factors is high but the communities are unaware of CVD - Community members are unable to travel for care or afford treatment after diagnosis - The nurses were also found to lack the relevant training and medication for treating CVD conditions such as hypertension - The participants emphasized the need for improving ancillary support for primary care operations |
| **Lamptey et al. (2017)** | Communities in Lower Manya-Krobo district, Eastern region | Urban | NCDs (Hypertension), management and control | Study design: Quasi-experimental design  Method: Quantitative | Community | Sample: 2400 participants  Age: 30 – 65 years | - The overall prevalence rate of hypertension was 32.4% (3.14% in control and 33.4% in intervention communities) - Overall awareness of hypertension was 46.2% (44.7% in control and 47.7% in intervention communities) - Overall percentage of treatment was 9% across both intervention and control - Overall hypertension control was 1.3% (0.5% in control and 2.1% in intervention communities) - Predictors of hypertension were age, increasing BMI, waist, and hip circumference - When age was controlled for, risk factors of hypertension included overweight and obesity - Awareness was higher among older adults than younger adults, and also among groups with family history of hypertension or CVDs |
| **Read et al. (2020)** | Communities in Accra in Greater Accra region, and Kintampo South in Bono East regions | Rural and urban | Mental illness, care and management | Study design: ethnographic and participatory  Method: Qualitative – interviews, observation, group meetings | Community | Sample: 27  Age = not stated | - Desire for work and earning sufficient money constituted important aspect of recovery and social inclusion - Participants encountered significant challenges in the areas of finding and remaining in employment - Participants who had jobs in the private sector also experienced discrimination in the workplace. - Participants in the public sector and unionized members have access to legal protection in the event of illness. - Existence of potential human rights legislations such as the Disability Rights Act, Labour Law and Mental Health act for improving recovery and social inclusion of individuals living with mental illness - Access to social protection programs such as Livelihood Empowerment Against Poverty (LEP) - Partnerships with institutions such as Commission on Human Rights and Administrative Justice (CHRAJ), Mental Health Authority, (MHA) NGOs, National Council on Persons with Disability (NCPD) |
| **Ojo et al (2020)** | Communities in La Dade-Kotopon Municipal Assembly, Greater Accra | Urban | NCDs (Stroke), prevention | Study design: Multi-method qualitative study  Method: Qualitative – focus group discussion | Community | Sample size = 38  Age = not stated | - The DESERVE intervention was found to be a good fit across all the stakeholders - Elements of the intervention that were found to be transferable were; (i) focus on risk knowledge, medication adherence and patient-physician communication, (ii) facilitations by lay workers, (iii) use of patient testimonials, (iv) use of spirituality framework, (v) application of community-based approach - Facilitators of implementation included; desire to care for self and children, self-efficacy and intervention coherence - Potential barriers included; normalization of spiritual aetiology of illness, expectation of treatment on the spot of screening, physicians not seeming themselves as best implementers, implementation cost, feasibility of intervention |
| **Agongo et al, 2021** | Community engagement and feedback of results in the H3Africa AWI-Gen project: Experiences from the Navrongo Demographic and Health Surveillance site in Northern Ghana | Rural | Obesity and cardiometabolic disease | Prospective cohort studies/longitudinal | Community | Illness management and control    N: Not mentioned in paper | - The team observed from their interaction with the community that participants were overly expectant with regard to feedback activities. - Some participants thought the test results would be given back to them within a short time frame and they had the impression that those needing treatment would have the cost borne by the NHRC. - Several recommendations were made. This includes giving participants immediate feedback, having activities at an appropriate time, the location of engagement activities is important . - Overall community engagement processes and experiences highlight the importance of incorporating engagement as an integral part of the research process |
